# Pulsed Eddy Current Sensing for Critical Pipe Condition Assessment

**DOI:** 10.3390/s17102208

**Published:** 2017-09-26

**Authors:** Nalika Ulapane, Alen Alempijevic, Teresa Vidal Calleja, Jaime Valls Miro

**Affiliations:** Centre for Autonomous Systems (CB 11.09.300), Faculty of Engineering and Information Technology, University of Technology Sydney, 15 Broadway, Ultimo, NSW 2007, Australia; Alen.Alempijevic@uts.edu.au (A.A.); Teresa.VidalCalleja@uts.edu.au (T.V.C.); Jaime.VallsMiro@uts.edu.au (J.V.M.)

**Keywords:** critical pipes, eddy currents, Gaussian process, NDE, NDT, PEC, sensors

## Abstract

Pulsed Eddy Current (PEC) sensing is used for Non-Destructive Evaluation (NDE) of the structural integrity of metallic structures in the aircraft, railway, oil and gas sectors. Urban water utilities also have extensive large ferromagnetic structures in the form of critical pressure pipe systems made of grey cast iron, ductile cast iron and mild steel. The associated material properties render NDE of these pipes by means of electromagnetic sensing a necessity. In recent years PEC sensing has established itself as a state-of-the-art NDE technique in the critical water pipe sector. This paper presents advancements to PEC inspection in view of the specific information demanded from water utilities along with the challenges encountered in this sector. Operating principles of the sensor architecture suitable for application on critical pipes are presented with the associated sensor design and calibration strategy. A Gaussian process-based approach is applied to model a functional relationship between a PEC signal feature and critical pipe wall thickness. A case study demonstrates the sensor’s behaviour on a grey cast iron pipe and discusses the implications of the observed results and challenges relating to this application.

## 1. Introduction

Pulsed Eddy Current (PEC) sensing is considered as the most versatile member of the family of Eddy Current (EC) Non-Destructive Evaluation (NDE) techniques. PEC signals are known to possess a broad frequency spectrum enabling them to penetrate into different depths and to provide information about the geometry of the test piece being evaluated [1]. This capability has made PEC sensing a popular NDE technique for ferromagnetic material inspection in research and commercial domains [2,3,4,5]. Critical pipes are large diameter (usually ≥300 mm) pressurized pipes owned and managed by water utilities throughout the world for the purpose of distributing consumable fresh water to customers. Globally, most urban water utilities have extensive large, critical pressure pipe systems, parts of which have been in service for over a century [6,7,8]. Since these aged pipes are found in the form of three ferromagnetic materials, namely grey cast iron, ductile cast iron and mild steel, NDE of these pipes by means of electromagnetic sensing is a necessity. Water utilities undertake NDE to ascertain the structural integrity of their assets and make crucial decisions on pipe maintenance and renewal to prevent costly and catastrophic pipe failures. Motivated by this need an approach to effectively employ PEC sensing for NDE of critical pipes is presented and the underlying challenges related to the application are discussed.

The work in [9] provides a comprehensive review of state-of-the-art inspection technologies used for condition assessment of pipes. Provided that water utilities drive towards estimating stress concentration on pipe walls in order to predict failures, the critical piece of input information required for that is a dense map of the remaining pipe wall thickness [10]. PEC sensing therefore has higher preference for this application as it is capable of directly measuring the remaining thickness of healthy material and producing dense maps while exhibiting low sensitivity to tuberculation formed on aged critical pipes in the form of corrosion and graphitisation. In addition, it is not sensitive to insulated protection present in the form of internal cement lining should sensing be undertaken from inside the pipe [11,12,13]. Since critical pipe materials are ferromagnetic, recent works investigating PEC sensing of ferromagnetic material thickness [2,3,4,5] closely relate to the work of this paper. A commonality in those works is using the concentrically-wound exciter-detector coil-based PEC sensor architecture, which has been deemed to have better sensitivity for ferromagnetic material thickness quantification. Although there are other sensor architectures having magnetic sensors for signal reception as in [14,15,16,17,18], they have typically been used on non-ferromagnetic materials. Recent work in [19,20] has reported preliminary work explicitly on ferromagnetic pipe inspection. The work in [19] introduces a novel sensor design integrated with a distributed EC inspection system, while [20] presents a study of applying pulsed remote field eddy currents for internal inspection of pipes. Those techniques focus on defect detection and have produced promising results. However, they require further work to be able to deliver quantified remaining wall thickness in the form of dense maps, which is the critical piece of information demanded by water utilities.

Deriving from the work of [2,3,4,5], this paper exploits the exciter-detector coil-based PEC sensor architecture to inspect critical pipes by estimating remaining wall thickness using a time domain signal decay rate-based feature. The signal feature is derived from the analytical model proposed in [3] for PEC signals resulting from this architecture. Although [2,3,4,5] have analysed PEC sensor performance on standard steels such as Q235 and A3 under controlled experiments, their work on applications relating to critical pipe inspection is lacking.

Work specifically related to critical pipe inspection can be found in [11,21]. A study of the design of a PEC sensor that achieves signal sensitivity to grey cast iron thickness is presented in [11]. However, in [11], the sensor’s performance was not evaluated on actual pipes. On the other hand, [21] studies the use of the Gaussian Process (GP) [22] to infer grey cast iron pipe wall thickness by exploiting multiple features extracted by averaging regions of the signals produced by a commercial PEC sensor. The applicability of the results in [21] is limited to the specific commercial sensor. Although these two references form the basis for this paper, the main contribution here is the in-depth study of general PEC sensing applied to critical pipe assessment. More specifically, it studies the electrical and magnetic properties of a critical pipe material, and based on the quantification of these properties it proposes the sensor design, sensor excitation, signal acquisition and calibration strategies suitable for such materials. Finally, it proposes a GP-based approach to model the functional relationship between a PEC signal feature and material thickness, which is suitable for critical pipe wall thickness prediction.

The outline of this paper is as follows: Section 2 contains the theoretical formulation, which presents the PEC sensor operating principles with respect to ferromagnetic material thickness estimation, and the GP-based approach to model the functional relationship between a signal feature and pipe wall thickness. Section 3 presents PEC sensor design principles with particular reference to grey cast iron pipe assessment. Section 4 presents the experimental evaluation of the designed sensor’s performance. A case study depicting the sensor’s performance when using GP to estimate wall thickness of a grey cast iron pipe is also presented. Section 5 concludes this paper by discussing the implications of observed results and summarising viable practices and challenges for exploiting PEC sensing for state-of-the-art condition assessment of critical pipes.

## 2. Theoretical Formulation

### 2.1. Exciter-Detector Coil-Based PEC Sensor Operating Principle

**Figure 1:**
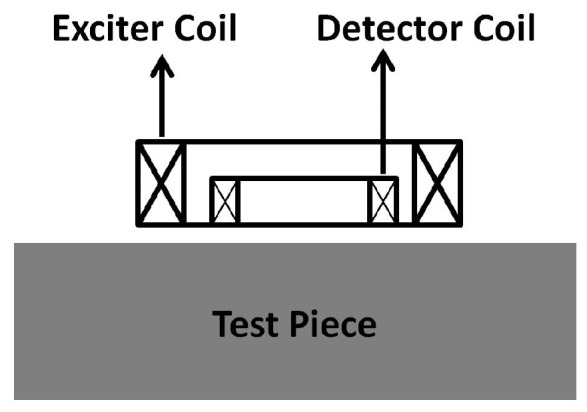
Cross-sectional view of a typical exciter-detector coil-based PEC sensor.

**Figure 2:**
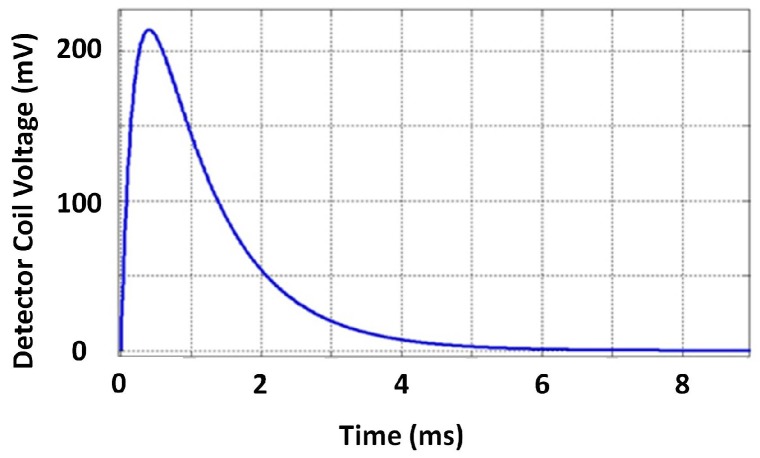
The typical shape of a PEC signal: induced voltage in the detector coil.

The typical exciter-detector sensor architecture sensitive to ferromagnetic material thickness, as exploited in this paper and in the ferromagnetic material thickness quantification-related works [2,3,4,5], is composed of two concentrically-wound, air cored, conductive circular coils, as shown in Figure 1. Rarer, though also practical, is the use of concentrically-wound rectangular coils [12,21]. In both configurations, one coil behaves as the exciter, while the other acts as the detector, which captures the signal. The exciter coil is excited with a voltage pulse that can theoretically be modelled as a Heaviside step function. The pulsed excitation causes a rapid change in the surrounding magnetic field; this in turn induces eddy currents in the test piece being assessed. The net effect of induced eddy currents and the excitation pulse induces a unique time-varying voltage in the detector coil. It is this detector, the coil voltage, that is identified as the PEC signal, which carries information about the test piece. The typical shape of such a PEC signal is an exponential decay as shown in Figure 2.

As done in [3], the decaying part of the time (*t*) domain PEC signal V(t) can be modelled as an infinite summation of exponential terms as shown in Equation (1) where the $b_{i}$ and $c_{i}$ terms are constants containing information about the properties of the sensor and the ferromagnetic test piece.

(1) $$V\left(t\right)=\sum_{i=1}^{\infty }b_{i}\exp(-c_{i}t)$$

All $c_{i}\geq 0$, $c_{i}\neq c_{j}$ when i≠j for i,j∈N.

To derive the signal feature used in this paper, we express Equation (1) in its logarithmic form as shown in Equation (2).

(2) $$\ln\left[V(t)\right]=\ln\left[\sum_{i=1}^{\infty }b_{i}\exp(-c_{i}t)\right]$$

Since all $c_{i}\geq 0$, Equation (1) becomes a sum of exponential decays. Therefore, we consider the later stage of the signal (i.e., t>>0) and apply the properties of the log sum of exponentials [23] to Equation (2). For the region t>>0, Equation (2) can hence be reduced to the dominant exponential as:

(3) $$\ln\left[V\left(t\right)\right]{|}_{t>>0}\approx \ln\left[b_{1}\exp\left(-c_{1}t\right)\right]$$

where $c_{1}$ is the dominant time constant and $b_{1}$ is the corresponding coefficient of the exponential term. Expanding the right-hand side of Equation (3) results in:

(4) $$\ln\left[V\left(t\right)\right]{|}_{t>>0}\approx -c_{1}t+\ln\left[b1\right].$$

We now define the signal feature β by taking the derivative of Equation (4).

(5) $$\frac{d\ln[V(t)]}{dt}{|}_{t>>0}\approx -c_{1}$$

(6) $$\beta =\frac{1}{c_{1}}$$

Equation (4) suggests that the behaviour of the later stage of a PEC signal taken on a ferromagnetic material should approximate a straight line with a negative gradient when expressed in its logarithmic form. Numerical simulations and experimental results in the subsequent sections validate this approximation. The feature β is the reciprocal of the absolute gradient of the logarithmic signal region behaving as a straight line. In return, β extracts the dominant time constant $c_{1}$, which dictates the later stage of the signal V(t) expressed in Equation (1). Previous work [4] has noted that this dominant time constant behaves as $c_{1}\propto 1/\left(\mu \sigma d^{2}\right)$ where μ, σ and *d*, which are magnetic permeability, electrical conductivity and thickness of the inspected ferromagnetic material under the domain of influence of the sensor, respectively. Since this behaviour has been reported on flat plates and the focus of this work is pipe walls, which are curved, the behaviours for large diameter pipes and the corresponding sensor geometry are verified through Finite Element Analysis (FEA) in [12], and it has been observed that the critical pipe surfaces behave the same way as flat plates due to the curvature being low with respect to sensor dimensions. Thus, β behaves as $\beta \propto \mu \sigma d^{2}$ for large diameter pipes. Therefore, when calibrated to a particular material, β reduces to a function dependent on thickness alone, making it possible to use the inverse of this function for thickness quantification. Furthermore, simulated and experimental results in this paper suggest that β has a desirable quality of having low sensitivity to lift-off, making β a suitable option for critical pipe inspection applications where unknown lift-off is a prevalent challenge. The non-linearity of β is particularly prevalent in lower and higher ends of thickness due to limitations in how high or how low the excitation strength delivered by sensor driving electronics can be, suggesting that a non-linear, potentially non-parametric modelling technique would be suitable for the application.

### 2.2. Gaussian Process Formulation

As established in Section 2.1, once calibrated for a material (i.e., for a particular material having properties μ and σ), thickness *d* reduces to a non-linear function dependent on β alone. Therefore, estimating thickness from PEC sensor signals can be formulated as a non-linear regression problem. Gaussian process models are a powerful tool to solve such regression problems.

Given the set of β values extracted from PEC signals $B={\left[\beta_{1},\beta_{2},\ldots ,\beta_{m}\right]}^{T}$, where each $\beta_{i}$ is associated with a noisy value of thickness $d_{i}$ in the set $D={\left[d_{1},d_{2},\ldots ,d_{m}\right]}^{T}$, the aim is to find the underlying function that maps β values to actual thickness. GP is used to learn the thickness distribution and to predict this distribution for arbitrary points $\beta^{*}$. In this paper, the training dataset [B,D] is produced through numerical simulation as explained in Section 4.4.

To apply the GP framework to this regression problem, a kernel K(B,B) whose elements are given by $k_{i,j}=k\left(\beta_{i},\beta_{j}\right)$ has to be selected. After evaluating a number of commonly used kernels, the squared exponential kernel was chosen for this work as it was found to be effective. This kernel is given by:

(7) $$k\left(\beta_{i},\beta_{j}\right)=\alpha^{2}\exp\left\{-\frac{1}{2\eta^{2}}{\left(\beta_{i}-\beta_{j}\right)}^{2}\right\}.$$

where α and η are hyper-parameters. These hyper-parameters together with noise standard deviation $\sigma_{n}$ are learned from the training data, by minimizing the negative log marginal likelihood:

(8) $$-\log p\left(D|B,\theta \right)=\frac{1}{2}D^{T}\Sigma^{-1}D+\frac{1}{2}\log\left|\Sigma \right|+\frac{m}{2}\log\left(2\pi \right)\;$$

where the covariance function Σ is given by:

(9) $$\Sigma =K\left(B,B\right)+\sigma_{n}^{2}I\;$$

with respect to $\theta =\{\alpha ,\eta ,\sigma_{n}\}$, where *I* is the corresponding identity matrix.

Once hyper-parameters θ are learned from the training data, arbitrary points $\beta^{*}$ are provided as input to the GP model to predict the corresponding thickness estimate as a Gaussian probability distribution whose mean is $\mu_{d}^{*}$ and standard deviation is $\sigma_{d}^{*}$. $\mu_{d}^{*}$ and $\sigma_{d}^{*}$ are calculated as follows:

(10) $$\mu_{d}^{*}=K\left(\beta^{*},B\right)\Sigma^{-1}D;$$

(11) $${\left(\sigma_{d}^{*}\right)}^{2}=\alpha^{2}+\sigma_{n}^{2}-K\left(\beta^{*},B\right)\Sigma^{-1}K\left(B,\beta^{*}\right).$$

## 3. A Sensor Design Example

This section details the procedure for designing an exciter-detector coil-based PEC sensor suitable for critical pipe inspection. The design example targets grey cast iron pipe assessment; the maximum thickness expected on pipes was 20 mm. The procedure includes the following steps: (1) identifying electrical and magnetic properties of the pipe material to be inspected; (2) numerically simulating a sensor to determine suitable sensor dimensions; and (3) sensor fabrication. The following subsections detail the three sub-steps of the design procedure.

### 3.1. Identification of Material Electrical and Magnetic Properties

Since β becomes a function in the form of β≈g(μ,σ,d) and predominantly depends on the electromagnetic properties and material thickness, it is in fact heavily independent of sensor dimensions. Practical limitations in sensor excitation and signal acquisition electronics dictate those dimensions, and they should be decided upon before fabrication in order to achieve sufficient penetration depth in the ferromagnetic material (grey cast iron in this case). Sensor dimensions are determined in this paper through numerical simulation, and to achieve that, knowing μ and σ beforehand is necessary.

Measuring electrical and magnetic properties was done by extracting a coupon (hot tapping [24,25,26] is a viable option for coupon extraction from on-site critical pipes), making a specimen (dimensions = 3 mm × 2 mm × 2 mm) through Electric Discharge Machining (EDM) wire cutting [27,28,29] (using cooling liquid) and feeding it to a Physical Property Measurement System (PPMS) [30,31,32]. An average representation of properties is derived by performing measurements on multiple specimens. A total of 27 specimens made from coupons extracted from equally-spaced locations along a 1 km-long grey cast iron pipeline, with details provided in Table 1, were tested by measuring their magnetization curve (i.e., BH curve where B is magnetic flux density and H is magnetic filed intensity) and electrical conductivity.

**Figure 3:**
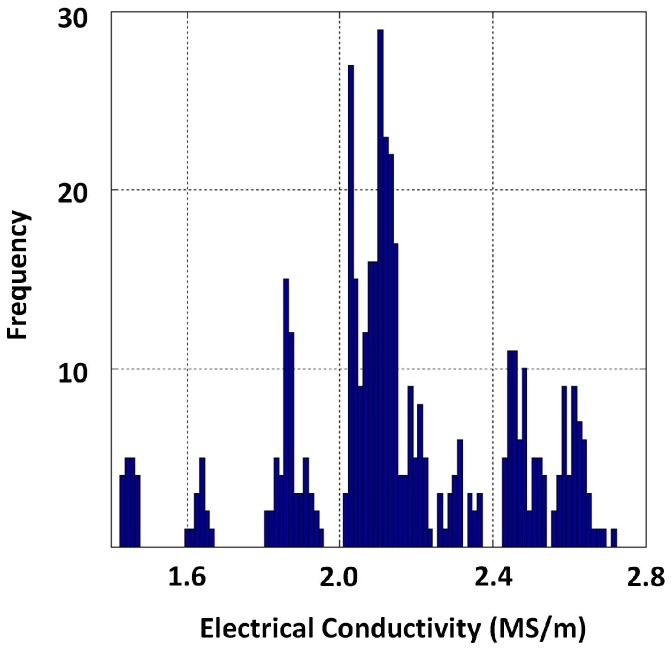
Histogram of the electrical conductivity of grey cast iron for temperatures between 283 K and 313 K captured.

Since electrical conductivity is known to vary significantly with temperature, the dependence was captured by measuring the conductivity of each specimen across a range (220 K–350 K). The average representation for conductivity (i.e., 2.16 $\times \;10^{6}$ S/m, approximately) was obtained by computing the mean over temperature (between 283 K and 313 K to resemble atmospheric temperature variation in Sydney Australia), as well as specimens. Although the average value of σ was considered for simulation, it was notable that σ of cast iron is considerably variable. This can be seen in Figure 3, which shows a histogram of conductivities resulting from all conductivity values (1097 in total) between temperatures 283 K and 313 K captured from all 27 specimens. The standard deviation (std) of this dataset was $0.261\times 10^{6}$ S/m, and 94.8% of the data fell within ±2 standard deviations. Such a variation in conductivity creates a unique difficulty in calibrating PEC sensors for critical pipe assessment. Conductivity data from which the statistics were calculated are provided as Supplementary Material.

**Figure 4:**
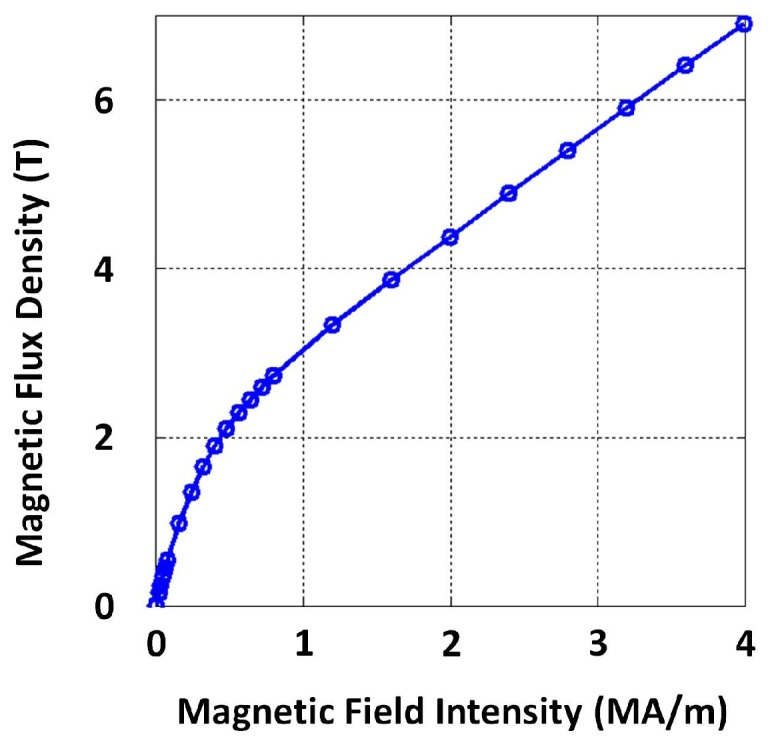
A measured magnetization curve of a specimen taken from a grey cast iron pipe segment.

**Figure 5:**
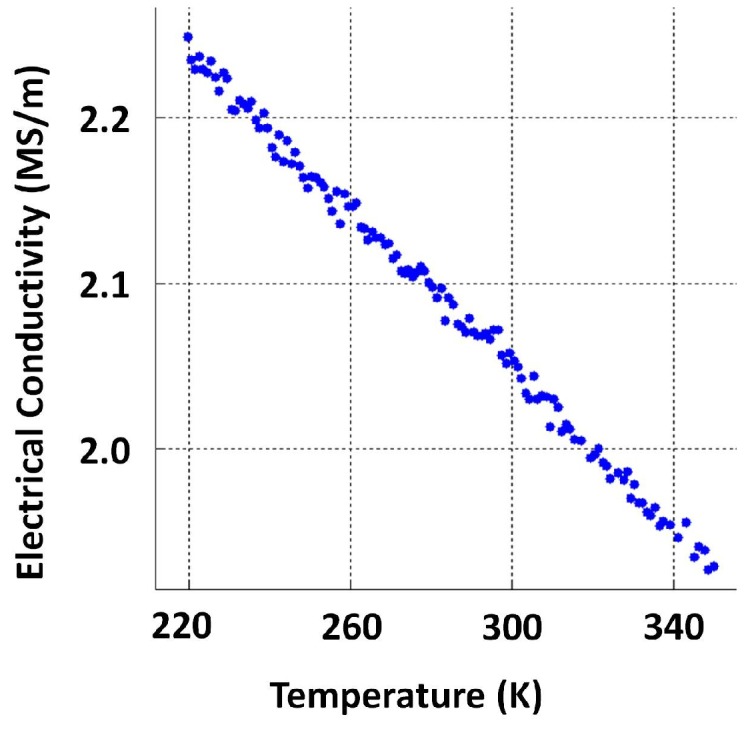
Temperature variation of the electrical conductivity of grey cast iron.

Significant variation in magnetic properties within atmospheric temperature conditions and a correlation between electrical conductivity and magnetic permeability were not evident from the available data; therefore a magnetization curve per specimen was measured while magnetizing and demagnetizing. Resulting curves were averaged eventually across samples. Figure 4 and Figure 5 depict a magnetization curve and conductivity measurements performed on a particular grey cast iron specimen, respectively, and raw data are provided as Supplementary Material. A fine sampling resolution to measure the magnetization curve region covering low magnetic fields is recommended in order to capture the typical non-linear behaviour present. A sampling interval of 10 A/m was used when magnetic field intensity ≤100 A/m. The relative permeability value $\mu_{r}=63$ calculated from the low magnetic field region of the magnetization curve in Figure 4 (considering the sensor excitation strength, high magnetic fields are not expected inside the pipe material) and the averaged conductivity value $2.16\times 10^{6}$ S/m were used to numerically simulate a PEC sensor and determine suitable dimensions for the application, the process is described in subsequent Section 3.2. 

### 3.2. Numerical Simulation of the PEC Sensor

**Figure 6:**
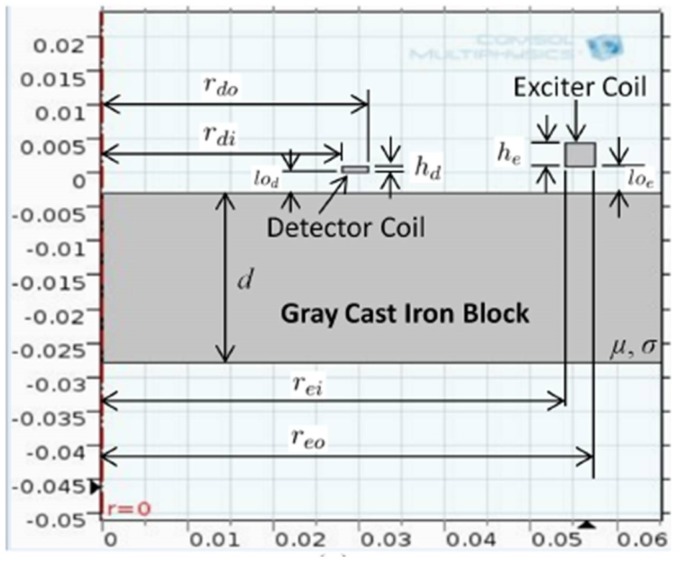
2D axisymmetric model of the PEC sensor placed on a cast iron block (developed in COMSOL Multiphysics^®^).

Due to the simplicity of modelling, and the common use for ferromagnetic material thickness estimation [2,3,5], a circular-shaped PEC sensor having concentrically-wound air cored coils as shown in the cross-section in Figure 1 was selected for this work. For a fixed excitation, the sensor size has been observed to be a dominant factor influencing the sensor’s penetration capability (i.e., the maximum thickness of a particular material to which the sensor will be sensitive) [16]. Therefore, before fabrication, the sensor interaction with grey cast iron was numerically simulated using FEA. A 2D axisymmetric model of the sensor placed above a grey cast iron (shown in Figure 6) was developed using COMSOL Multiphysics^®^. The input parameters required for simulation are defined in Table 2. The simulation model outputs the detector coil voltage V(t) as a function of many input variables as shown in Equation (12). V(t) is calculated using the magnetic vector potential, which is determined by solving the magnetic vector potential equation shown in Equation (13) for any given location in the model, where $\vec{A}$ is the magnetic potential at any location, *t* is time and ${\vec{J}}_{s}$ is the source current.

(12) $$V\left(t\right)=f\left(r_{di},r_{do},h_{d},lo_{d},n_{d},\sigma_{d},\mu_{d},r_{ei},r_{eo},h_{e},lo_{e},n_{e},\sigma_{e},\mu_{e},d,\mu ,\sigma ,Z_{dl},I_{e},t\right)$$

(13) $$\nabla^{2}\vec{A}-\mu \sigma \frac{\partial \vec{A}}{\partial t}=-\mu {\vec{J}}_{s}.$$

To narrow down the suitable set of sensor dimensions, the heights of exciter and detector coils ($h_{e}$ and $h_{d}$) and vertical offsets of the two coils ($lo_{e}$ and $lo_{d}$) are fixed. Copper wires are used to wind coils; thus, we use the standard permeability and conductivity of copper ($\mu_{e}$, $\mu_{d}$, $\sigma_{e}$ and $\sigma_{d}$) for simulation. In addition, rough estimates of permeability (μ) and electrical conductivity (σ) of grey cast iron are required. Table 3 shows the fixed parameters for simulation. As per the estimated value in Section 3.1, the approximated electrical conductivity used for grey cast iron was $\sigma =2.16\times 10^{6}$ S/m. Since the magnetic properties of grey cast iron are non-linear, the relative permeability value $\mu_{r}=63$ calculated from the low magnetic field region of the experimentally-measured BH curve in Section 3.1 was used to represent μ. The amplitude of the excitation current pulse was also considered to be fixed at 200 mA. The sensor excitation circuit was designed to produce a voltage pulse having a 10-V amplitude and current amplitude of 200 mA; thus, the exciter coil resistance was required to be $R_{e}=50$Ω. Due to availability, standard copper wire of $d_{e}=d_{d}=0.315$ mm diameter (AWG 28 wire class) was chosen to wind both exciter and detector coils. As discussed in Section 3.3, the detector coil output is directly connected to an instrumentation amplifier having high input impedance. This results in the effective impedance ($Z_{dl}$) felt as the load by the detector coil to be high (indicative of ∞).

**Figure 7:**
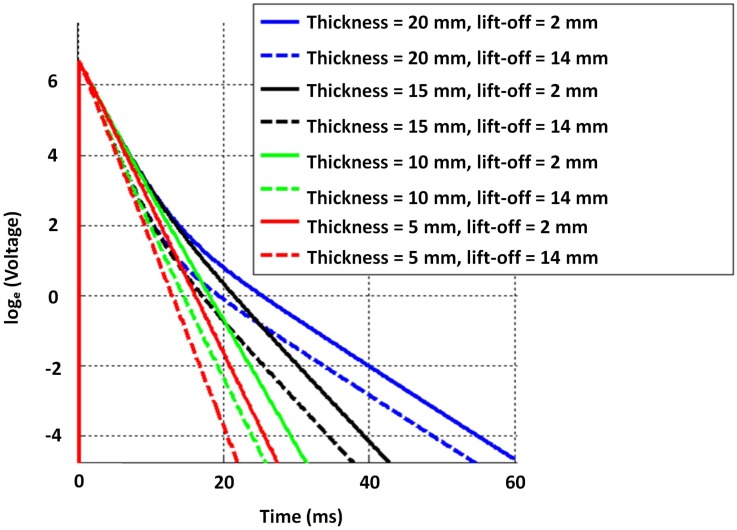
Numerically-simulated signals by the 2D axisymmetric model for different grey cast iron thickness with and without lift-off (visualized as ln[V(t)]); sharp rising edges of signals are not discriminable in the ms time scale and appear to be overlapping.

Given the constraints, the objective was to select suitable inner and outer radii of both exciter and detector coils (i.e., $r_{ei}$, $r_{eo}$, $r_{di}$ and $r_{do}$) along with their respective number of coil turns (i.e., $n_{e}$ and $n_{d}$) in order to have cast iron thickness sensitivity from about 5–20 mm. While parameter selection can be formulated as an optimisation problem, similar to the work in [19] related to a distributed EC inspection system, solving it for this case would require time-consuming stochastic optimisation due to deriving closed-form equations to perform a quicker convex optimisation being difficult. Therefore the parameters in Table 4 were selected through simulation and experimentally validated to yield sufficient sensitivity to thickness with both low and high lift-off, as shown in Figure 7. The later stage of all signals in Figure 7 behaves as a straight line with a negative gradient; this behaviour is expected as per the formulation in Equation (4) and validates the straight line behaviour theorized in Section 2.1. Raw data plotted in Figure 7 and the relevant COMSOL simulation model are provided with the Supplementary Material for interested readers to use and with which to experiment.

Due to operational practicalities, water utilities are interested in robotic tools, which can autonomously inspect pipes internally; a development related to the purpose is presented in [13]. As in the case with the pipe in Table 1, critical pipes usually have an insulated internal protection, typically made of cement. Therefore, when inspecting internally, it is necessary for PEC sensors to assess a pipe wall with a 10–15 mm lift-off. Motivated by this need, and for the sensor to be suitable for both external and internal inspection, the dimensions in Table 4 were chosen for the sensor to be sufficiently sensitive to thickness with a lift-off ranging from 2–14 mm, as indicated by the signals in Figure 7.

### 3.3. Sensor Fabrication

**Figure 8:**
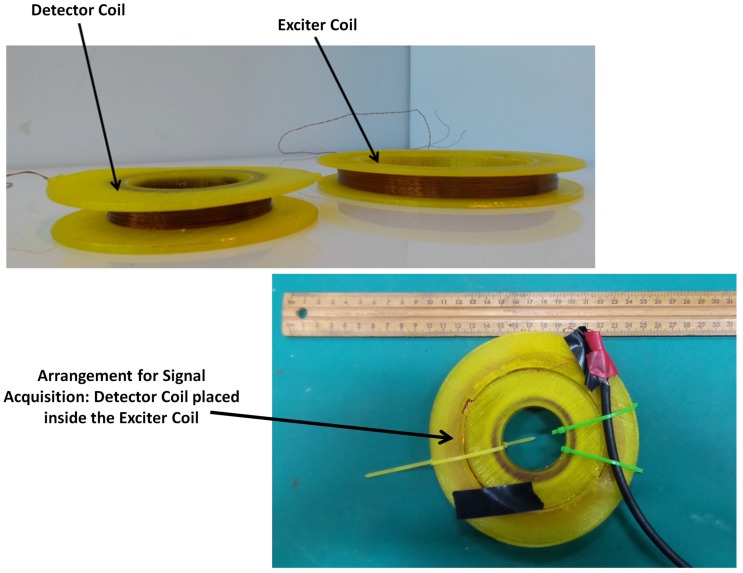
Fabricated PEC sensor.

Figure 8 shows the PEC sensor fabricated using the values provided in Table 4. Both exciter and detector coils were wound using AWG 28 wires having an approximate diameter of 0.315 mm. The sensor core was designed in SOLIDWORKS^©^software and was 3D printed using Polylactide (PLA) biodegradable polyester.

**Figure 9:**
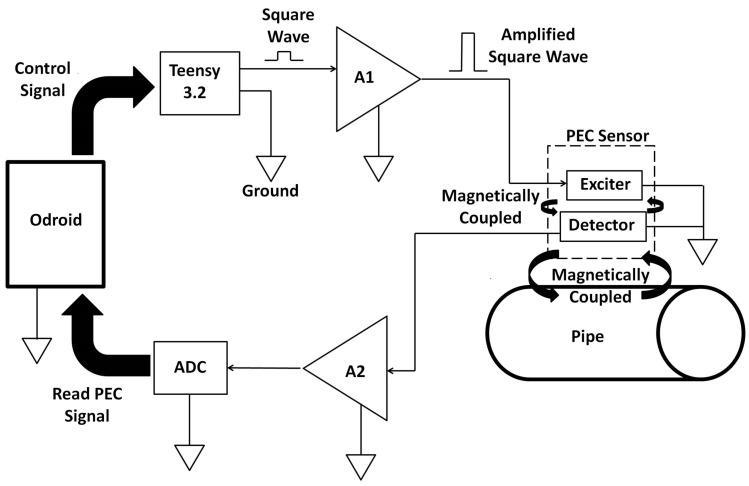
Block diagram of the PEC sensing setup.

**Figure 10:**
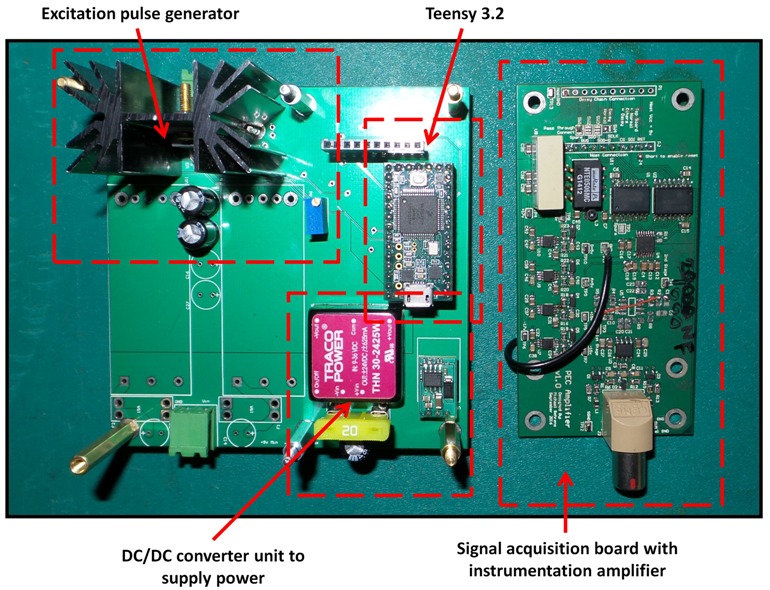
Fabricated PCB for sensor excitation and signal reception.

**Figure 11:**
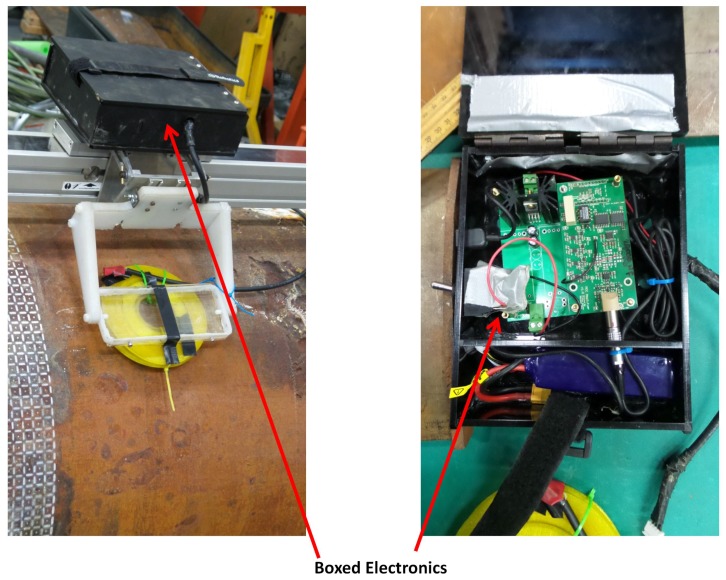
Sensing setup performing an external pipe scan.

The block diagram of the sensing setup composed by integrating the sensor with driving and receiving electronics is shown in Figure 9. An Odroid XU4 (Arm-based single board computer) is used to control a Teensy 3.2 generating the square wave (voltage pulse), which drives the sensor. The low power square wave generated by the Teensy is passed through an OPA548T fast Operational Amplifier-based non-inverting amplifier (A1) with a voltage gain of four to generate the desired powerful sensor excitation voltage pulse of 10 V, 50% duty ratio with a 60 ms pulse width. The induced detector coil voltage (i.e., PEC signal), which is in millivolt scale, is passed through an AD8428BRZ-based high performance instrumentation amplifier (A2) with a voltage gain of 2000. The amplified signal is sampled by a 16-bit ADS8681 integrated data acquisition system at a sampling rate of 15 μs per sample and transferred via the Odroid to a PC. Capturing multiple signals at a given location and considering their average as the measurement was applied for noise reduction. To be specific, following experimental observations, obtaining a measurement by averaging 10 signals resulting from falling edges of the pulse is implemented. Alternatively, the signal processing method in [33] using only one signal per measurement is also implemented in case quicker data acquisition is desired. The fabricated PCB for sensor excitation and signal reception is shown in Figure 10, while Figure 11 shows the sensing setup with cased up electronics being used to externally scan a pipe with the aid of an automated rig for sensor placement.

## 4. Experimental Work and Results

Experimental work done on grey cast iron to evaluate the designed sensor’s performance along with observed results are presented in the following subsections.

### 4.1. Sensitivity to Thickness

**Figure 12:**
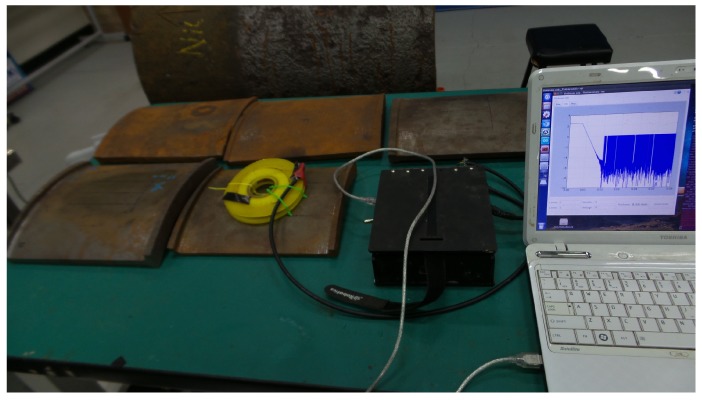
Experimental setup used with grey cast iron calibration blocks.

**Figure 13:**
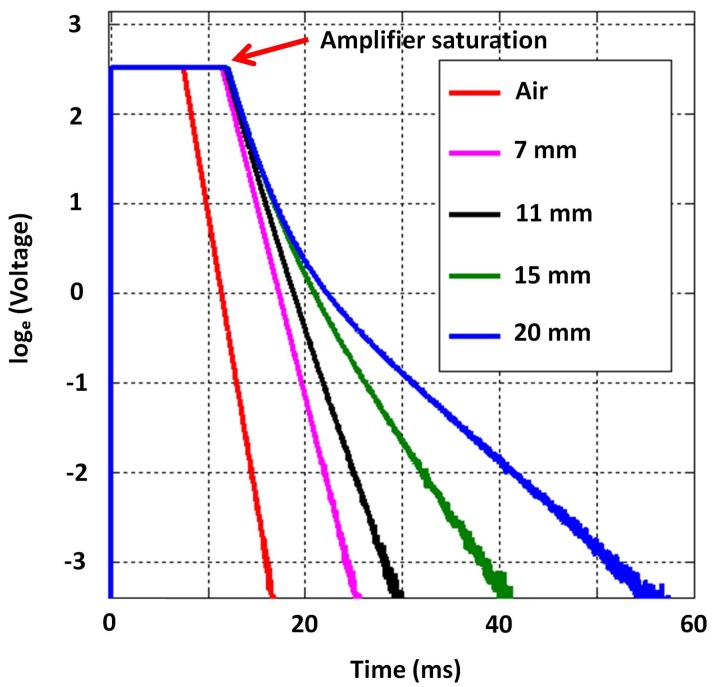
Signals obtained on grey cast iron calibration blocks for the full thickness range; sharp rising edges of signals are not discriminable in the ms time scale and appear to be overlapping.

First, the sensitivity to thickness of the designed sensor’s signals was evaluated. This was done by capturing signals on grey cast iron calibration blocks, which were machined from an on-site pipe exhumed from the pipe mentioned in Table 1. The experimental setup used is shown in Figure 12. The approximate length and width of a block was 300 mm and 200 mm, respectively, making them large enough to marginalise the edge effect on the signals. The first test was carried out by capturing signals on 7 mm, 11 mm, 15 mm and 20 mm block thickness values along with an air signal, shown in Figure 13. The expected straight line behaviour in the later stage of the signals is evident from the figure, as were the gradients, which translate to β in that region; therefore, experimentally validating that signals from the designed sensor were sensitive to grey cast iron thickness up to 20 mm.

**Figure 14:**
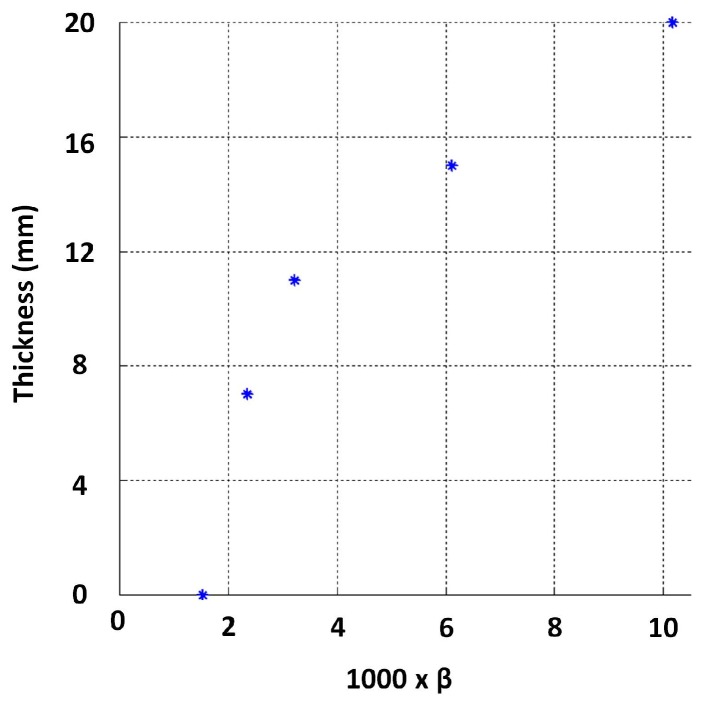
Functional behaviour between thickness and β obtained from calibration blocks.

Due to the ease of implementation, approximations for β values were obtained by line fitting through linear least squares to signal regions between −1 and −4 in the log scale. The behaviour between thickness and β observed for this dataset is shown in Figure 14. The relationship exhibits a clear functional behaviour, except in the low thickness region (i.e., thickness below 7 mm), needing further investigation.

### 4.2. Lift-Off Invariance

**Figure 15:**
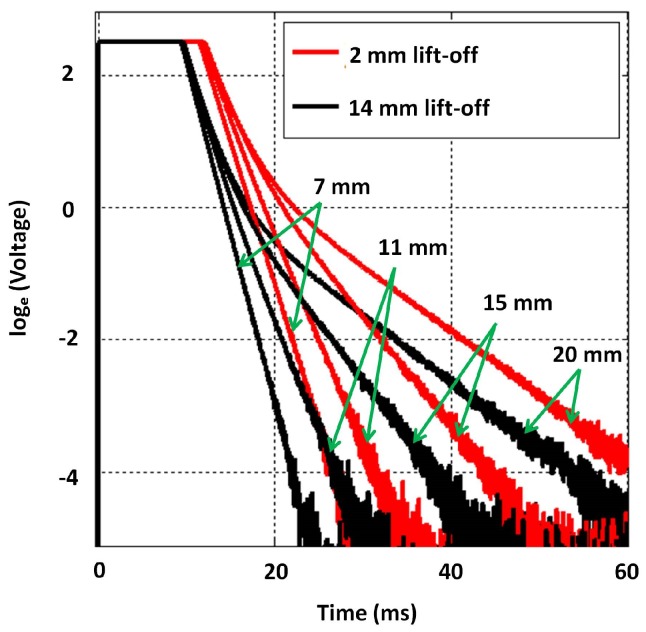
Influence of lift-off on signals across the full thickness range; sharp rising edges of signals are not discriminable in the ms time scale and appear to be overlapping.

While keeping the default excitation pulse amplitude at 10 V, the same selection of blocks was measured with an additional 12 mm lift-off introduced with perspex. It should be noted that the 2 mm lift-off is indicative of the sensor touching the block surfaces as this is the thickness of the sensor casing. Though the signals attenuate with increased lift-off, as per Figure 15, the gradient in the later stage remains largely invariant to the change. 

**Figure 16:**
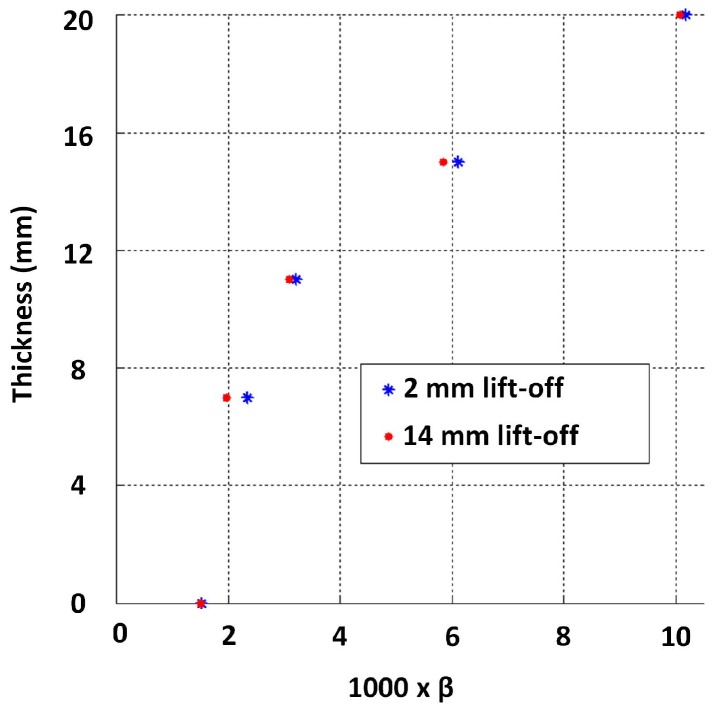
Influence of lift-off on the function between thickness and β.

As per Section 4.1, β was approximated by fitting lines to the signal regions between −1 and −4 for both 2 mm and 14 mm lift-off signals, and the relationship observed between thickness and those β values is shown in Figure 16. The influence of lift-off on β is evidently not significant, and a quantitative measure of the influence is presented in Section 4.4. This observation is consistent with notion in Section 2.1: β is dominantly influenced by μ, σ and thickness. In practice though, when lift-off is introduced, the PEC sensor’s magnetic field’s penetration capability is decreased due to the sensor moving away from the test piece. Provided flexibility to increase the excitation strength as lift-off increases, the lift-off’s influence on β should almost nullify completely.

### 4.3. Sensitivity to Low Thickness

**Figure 17:**
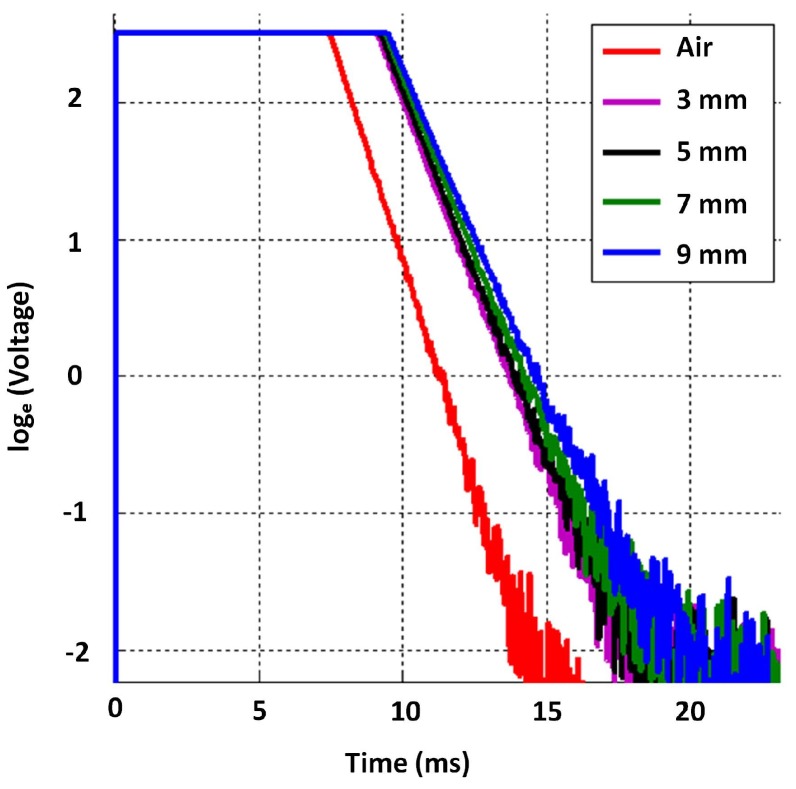
Sensitivity to low thickness at the default excitation amplitude (10 V) at 14 mm lift-off; sharp rising edges of signals are not discriminable in the ms time scale and appear to be overlapping.

In order to analyse sensitivity to low thickness, the sensor excitation voltage amplitude was kept at 10 V while signals were captured on grey cast iron blocks of thicknesses of 3, 5, 7 and 9 mm, at 14 mm lift-off. It is evident in the captured signals in Figure 17 that discriminating thickness in the low thickness region poses a challenge, and the gradients of all signals converge towards that of the air signal. This behaviour can be attributed to the PEC sensor in principle behaving as a current transformer. When the test piece is thin, the magnetic field reflecting from the induced eddy currents in the test piece can be much smaller than the driving magnetic field created by the currents flowing in the exciter coil. Therefore, the induced detector coil voltage is dominated by the direct coupling with the driving magnetic field, making it difficult to sense the much smaller influence created by the thin test piece. 

**Figure 18:**
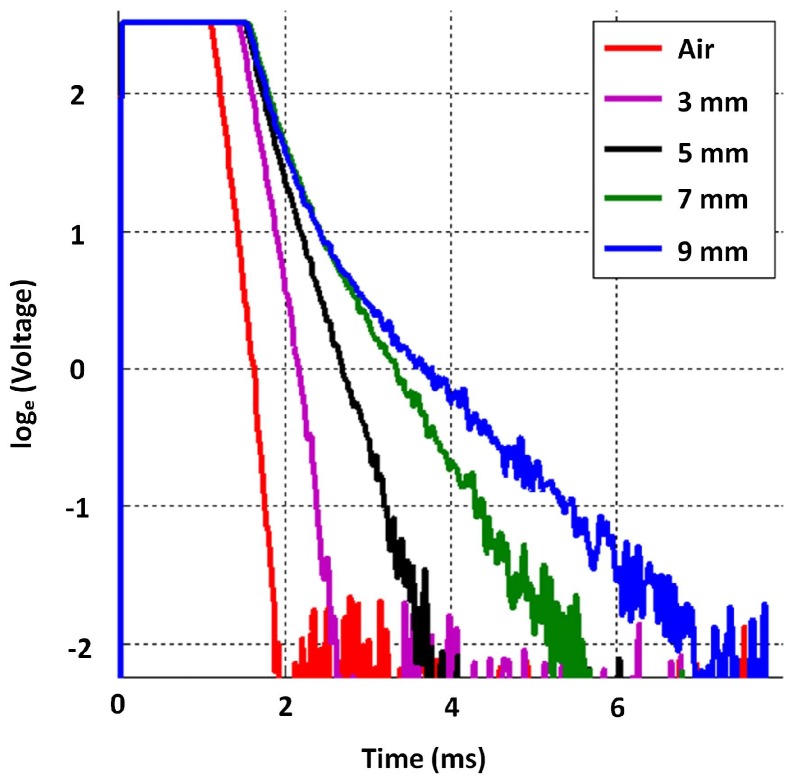
Sensitivity to low thickness at reduced excitation amplitude (3 V) at 14 mm lift-off; sharp rising edges of signals are not discriminable in the ms time scale and appear to be overlapping.

With the aim of achieving better sensitivity to low thickness, an experiment was conducted by reducing the excitation pulse amplitude on the same plates with the same lift-off of 14 mm. Discrimination within signals progressively improved as excitation amplitude decreased. When the pulse amplitude was reduced to 3 V, the discrimination evident in Figure 18 could be achieved. The reason behind this is the ratio between the magnitude of the reflected magnetic field created by eddy currents and the magnitude of the direct coupled magnetic field caused by the exciter coil current increasing. This happens as the direct coupled field reduces proportionally to the excitation current while the reflected field was not decreasing as much since the permeability of cast iron at such low magnetic fields was increasing due to the non-linear characteristic of the magnetization curve. 

**Figure 19:**
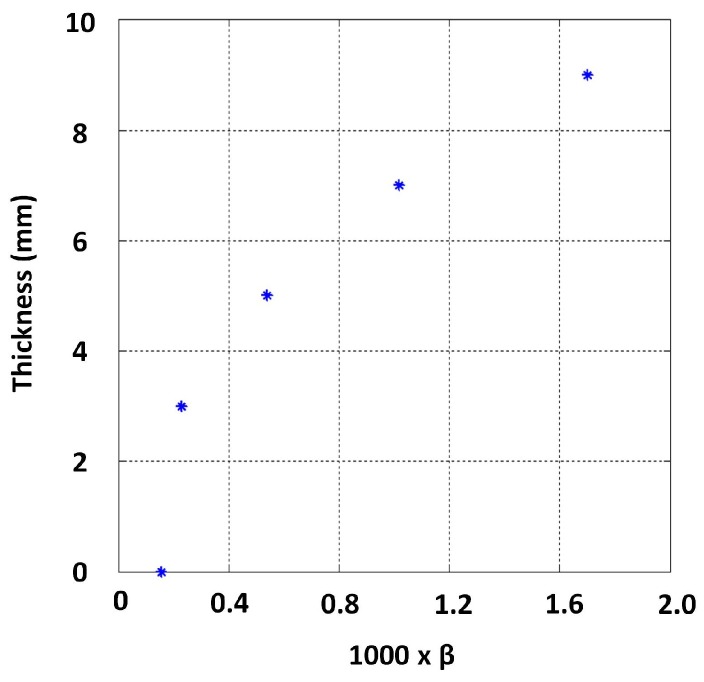
Experimentally-observed function between thickness and β at the low thickness region with reduced excitation strength and 14 mm lift-off.

**Figure 20:**
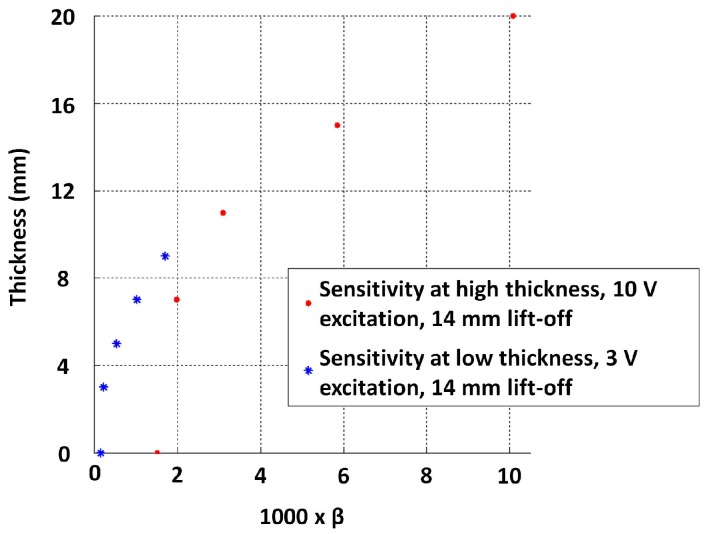
Low and high thickness regions captured with different excitation strengths merging to form a continuous function in the feature domain.

Lines were fitted to the log voltage scale interval between 0.5 and −1.5 to estimate β. The observed relationship between thickness and β is shown in Figure 19, with the exception of a much lower thickness range, i.e., less than 3 mm now. The variability to low thickness values along with the variability seen in the higher thickness region (11 mm and above) for the 14 mm lift-off case is shown in Figure 20. This observation suggests that a fixed voltage may not be suitable to discriminate a large thickness range. Further, reducing the excitation strength could increase precision in measuring lower thickness conditioned on sensor geometry being sufficiently large to create an appropriate magnetic field [16]. 

### 4.4. GP Modelling of the Function between Thickness (d) and β

**Figure 21:**
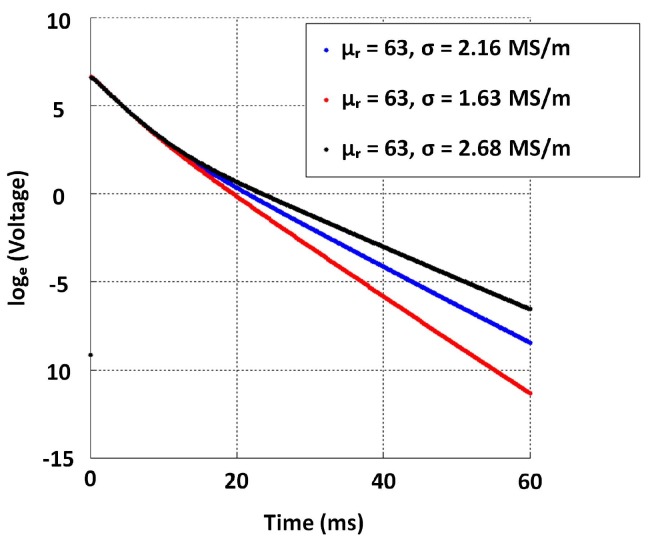
Signals simulated for 15 mm grey cast iron by varying electrical conductivity as per variation in conductivity observed in Section 3.1.

The purpose of this exercise was to model the relationship between thickness and β using GP for grey cast iron in order to predict unknown thickness when PEC signals are available. A persisting challenge though is the considerable variability of electrical conductivity of grey cast iron observed in Section 3.1 (mean σ=2.16 MS/m, std = 0.261 MS/m). This causes difficulty in calibration; therefore, we decided to simulate PEC signals using the numerical model proposed in Section 3.2 by varying the conductivity by two standard deviations about the mean since we observed close to 95% of conductivity values existing within a ± two standard deviation margin cantered about the mean. Therefore, we simulated 5, 10, ..., 30, 35 mm thickness values for the purpose of learning a function despite that the objective was for the sensor to be sensitive up to 20 mm, producing three signals on each thickness such that they correspond to conductivity values $\sigma_{min}=1.63$ MS/m, $\sigma_{max}=2.68$ MS/m and mean σ=2.16 MS/m, while $\mu_{r}=63$ remains. Figure 21 shows an example depicting signals obtained for a 15 mm thickness. All signals can be reproduced and studied by interested readers since we have provided the simulation model with the Supplementary Material.

**Figure 22:**
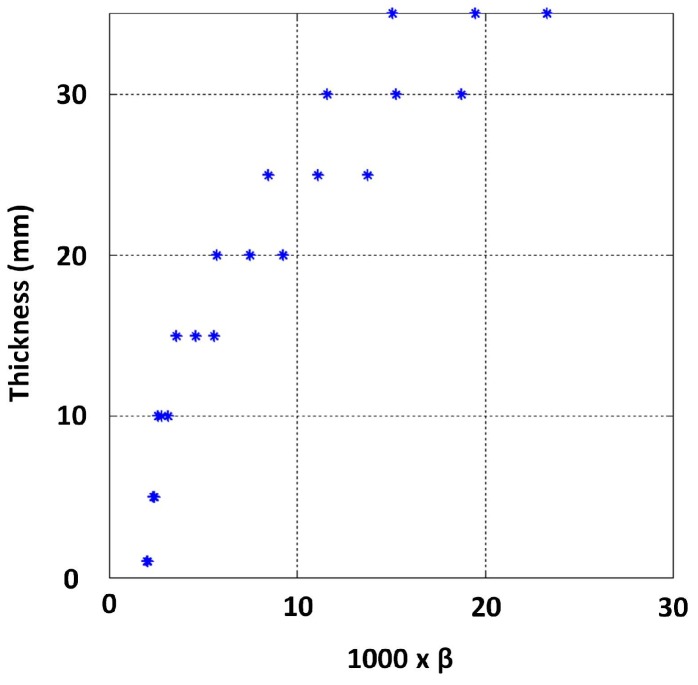
Effect of variation of σ on β.

**Figure 23:**
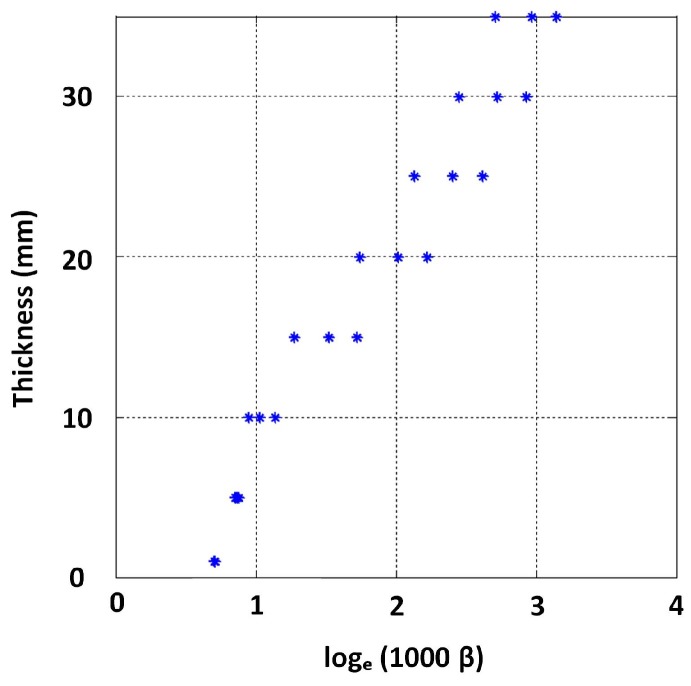
Influence of variation of σ on β reduced by expressing β in the logarithmic scale.

**Figure 24:**
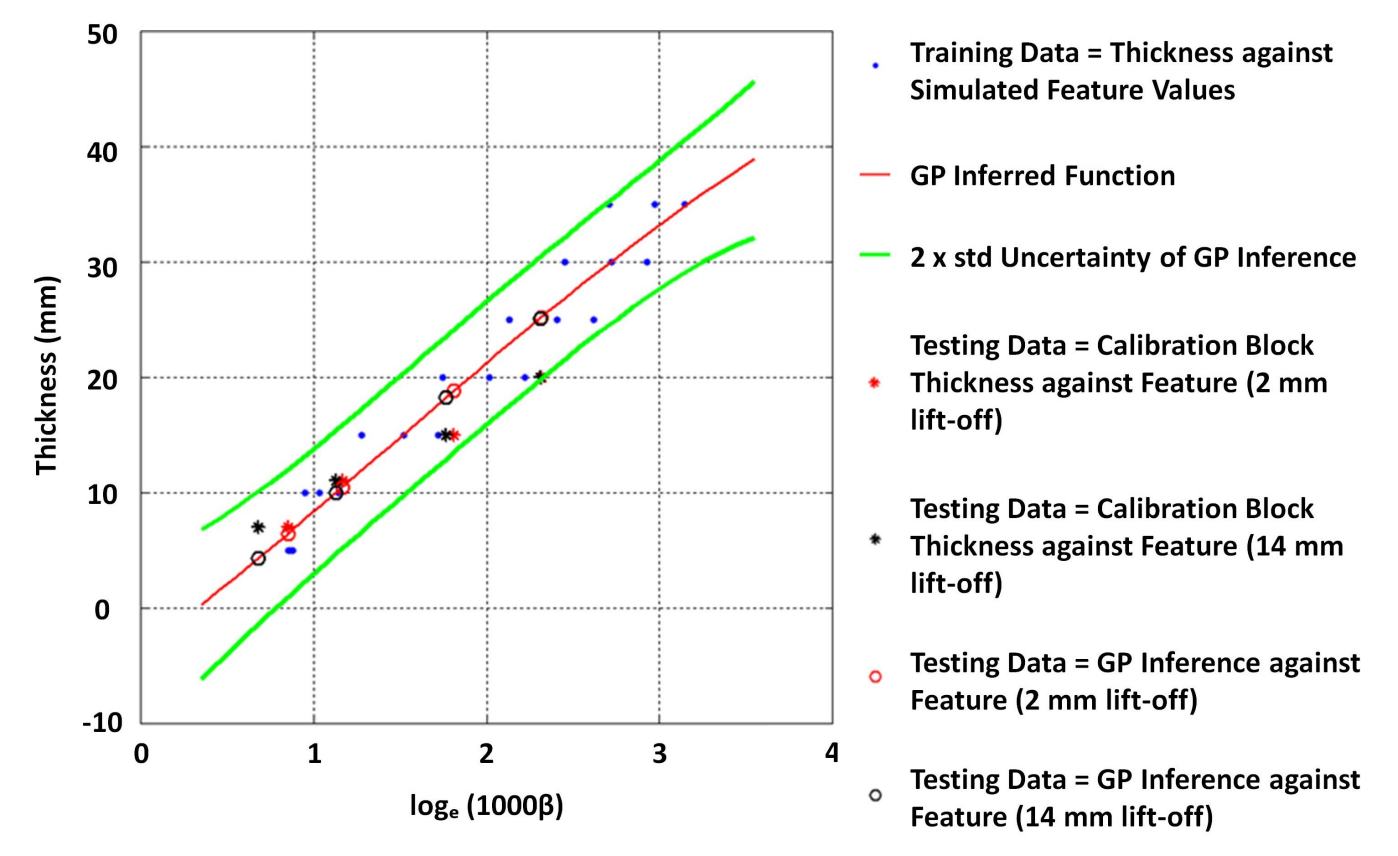
A GP modelled function usable to infer the unknown thickness from β with a considerable uncertainty.

β values form all the signals were approximated by fitting straight lines to the log voltage scale interval between −0.5 and −11.5. Figure 22 shows the resulting variability of β due to conductivity variation. The authors opted to model this relationship in its logarithmic form presented in Figure 23 by expressing β in log scale so that some linearity in the relationship becomes apparent and exploitable in modelling the function. By using all simulated β values for 5, 10, ..., 30 and 35 mm thickness values as training data, the stable GP model shown in Figure 24 was learned by minimizing the negative log marginal likelihood in Equation (8) as explained in Section 2.2. The code for learning the GP model is provided with the Supplementary Material.

The estimates and errors observed when estimating the thickness of available calibration blocks are shown in Table 5 and Table 6. The 2× std uncertainty on average for this model is about ±5.6 mm; this could be attributed to the variation evident in training β due to the inherent challenge of large variability in conductivity associated with grey cast iron and other similar critical pipe materials. The model was not trained for lower thickness values (i.e., less than 5 mm) since the objective was to exploit this model on real pipes, and such low thickness values are generally not expected. This model was used to assess wall condition of a grey cast iron pipe, which was part of the critical pipe asset introduced in Table 1, and the results are presented in Section 4.5.

It is thus noticeable that errors of about 5 mm can occur; however, the observed errors are close or slightly less than the anticipated 2× std error. 

### 4.5. A Case Study on a Grey Cast Iron Pipe

**Figure 25:**
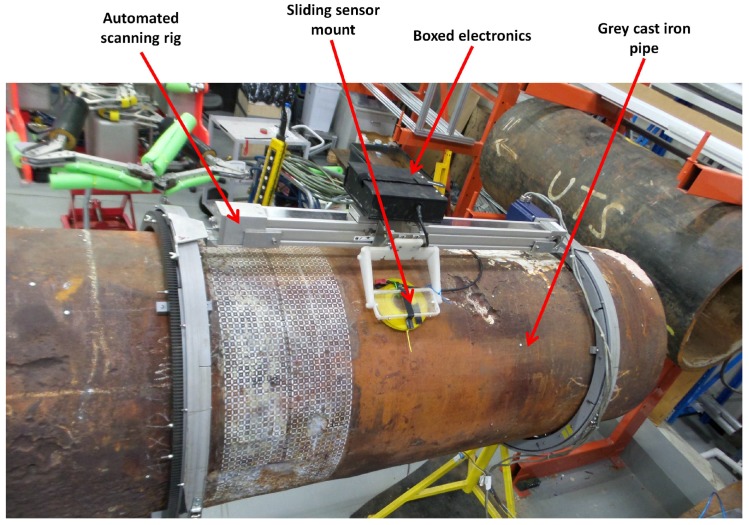
Experimental setup used to scan the grey cast iron pipe section using an automated pipe scanning rig.

The designed sensor was used to externally scan a grey cast iron pipe section exhumed from the pipe asset introduced in Table 1. The length of the scanned section was 650 mm, and the full pipe circumference of that section having an external pipe diameter of approximately 660 mm was scanned. The experimental setup used for scanning is shown in Figure 25. Measurements were taken with the aid of an automated pipe external scanner rig by translating the sensor in equal distance increments of 12.5 mm, both along the pipe axis, as well as along the circumference. Measurements were partially overlapping since $r_{eo}$ of the circular PEC sensor used is 57 mm. Although the thickness values estimated by the PEC sensor generalize to a larger area dictated by the sensor size, thickness values were plotted as a grid map having 12.5 mm × 12.5 mm resolution, which resulted in a dense, but smooth map. A total of 8400 measurements was taken and arranged as a grid map of size 168 × 50, i.e., 50 parallel rings that cover the full circumference and are perpendicular to the pipe axis have been measured with each ring containing 168 measurements. The distance between two adjacent measurements along a ring, as well as the distance between two adjacent rings was 12.5 mm.

Subsequently, the pipe was grit blasted to remove rust and graphitisation and laser scanned to obtain a 3D collocated point cloud of the pipe’s internal and external surfaces. The point cloud was up-sampled and ray traced as proposed in [34] to obtain a highly detailed Ground Truth (GT) representation of the remaining wall thickness map at a high resolution (GT is captured as a grid map with the distance between two nearest thickness values being 0.6 mm) in order visualize how PEC measurements correlate with the actual pipe condition.

Note that the PEC sensor has an exciter coil external diameter of 114 mm, and the resolution of the GT map is 0.6 mm. As the relationship between the thickness estimated by the PEC sensor and the actual pipe wall thickness at high resolution is not known, it is assumed that the sensor readings relate approximately to the average thickness under the exciter coil. Therefore, a qualitative comparison is done by averaging the GT thickness values falling within a circle of a 58 mm radius centred at every sensor location coordinate.

**Figure 26:**
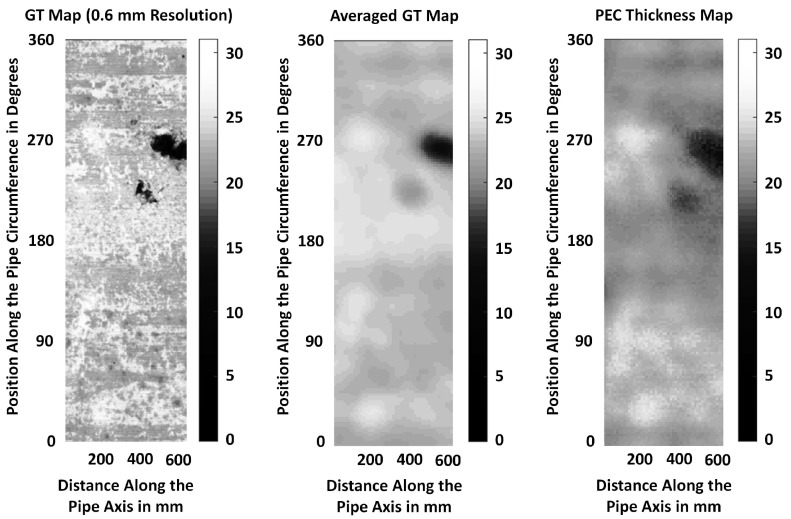
Thickness maps of a grey cast iron pipe.

Three maps showing estimated PEC thickness values, high resolution GT (at 0.6 mm) and averaged GT are presented on XY coordinate planes in Figure 26. In all three plots, the X-axis denotes the direction along the pipe, while the Y-axis denotes the direction along the circumference. Colour bars to the right of maps indicate wall thickness in mm. Distance along the axis is marked in mm, while the position along the circumference is marked in degrees. $360^{\circ }$ indicates full coverage of the pipe circumference. 

It is evident from the figures that the results produced by the designed PEC sensor show a correlation to the actual pipe condition and can produce a smoothed representation of actual thickness in the form of a dense map. It can be considered that the PEC map is marginally representative of the averaged, or down-sampled GT map. Further studies are required to learn the exact relationship that maps high resolution GT to PEC measurements along with quantification of likely errors.

## 5. Discussion and Conclusions

This paper presents advancements to PEC inspection in response to challenges encountered in the water industry sector. Operating principles of the exciter-detector coil-based sensor architecture with an associated sensor design and calibration strategy are presented. A log gradient-based feature is extracted, and a GP-based approach is employed to model the non-linear functional relationship between the feature value and pipe wall thickness. A case study demonstrates the sensor’s behaviour on a grey cast iron pipe wall thickness assessment.

Flexibility to vary excitation strength is discussed as a design consideration to overcome two opposing challenges. Foremost, the variable lift-off between the PEC sensor and the pipe asset was attributed to cement mortar lining, graphitisation or remanence of ground material; secondly, the interlinked eddy current penetration capability and signal saturation, which affect the precision of measuring lower or higher end thickness values.

This paper also studied and reported the variability of electromagnetic properties associated with critical pipe materials, specifically the electrical conductivity of grey cast iron. It was observed that the variability is large, and it poses a challenge by introducing high uncertainty (about ±5.6 mm) to thickness estimates produced through the GP-based approach proposed in this paper. This obstacle needs to be taken into consideration by water utilities and be accounted for in their decision making about pipe renewals when employing PEC for inspecting critical pipes, especially those made of cast irons. For the specific case of cast irons, provided the material inhomogeneity and the associated local variations of electrical and magnetic properties, normalizing the thickness estimates with respect to a selected thickness within a scan section may not completely nullify the challenge posed by material properties and, in some instances, may even increase errors.

Another challenge associated with PEC sensing is the limitation in spatial resolution, or sensitivity to the test piece condition localized to a point, or a small region. As evidenced in Figure 26, PEC measurements tend to reflect the condition of a region of the test piece rather that a localized point, and the region size tends to be dictated by the size of the sensor, hence limiting the sensor’s resolution. Reducing the sensor size may appear to be a simple solution to this challenge; however, reducing the sensor size also limits the sensor’s penetration capability [16], making sensor size reduction not so trivial. Therefore, one could also look at PEC signal deconvolution with the aid of the high resolution GT to retrieve thickness maps with higher spatial resolution.

The challenges with material properties and limited spatial resolution pave the way toward future work driving towards increasing PEC sensor resolution by making sensors smaller or otherwise while maintaining sufficient penetration capability and also solving the “inverse eddy current problem” of simultaneously estimating material thickness along with electromagnetic properties.

## Figures and Tables

**Table 1:** Specifications of the grey cast iron pipe used for the experimental work of this paper; adapted from [7].

Location	Verona Street, Strathfield NSW 2135, Australia
Year Installed	1922
Nominal Pipe Diameter	600 mm
Internal Pipe Diameter	579 mm–590 mm (with cement lining)
External Pipe Diameter	662 mm–666 mm
Nominal Wall Thickness	27 mm
Material	Pit cast iron
Internal Liner	Cement (installed in 1964)
Cement Lining Thickness	9.5 mm–16.5 mm
Pipe Lengths	3.6 m
Jointing	Lead run joints (with tar-soaked hemp sealants)
Total length in Use for Research	Approximately 1 km

**Table 2:** Parameters required for simulation.

Symbol	Description
$r_{di}$	Inner radius of detector coil domain
$r_{do}$	Outer radius of detector coil domain
$h_{d}$	Height of detector coil domain
$lo_{d}$	Vertical offset of the detector coil
$n_{d}$	Number of detector coil turns
$d_{d}$	Diameter of the detector coil wire
$\sigma_{d}$	Electrical conductivity of the detector coil
$\mu_{d}$	Magnetic permeability of the detector coil
$r_{ei}$	Inner radius of exciter coil
$r_{eo}$	Outer radius of exciter coil
$h_{e}$	Height of exciter coil domain
$lo_{e}$	Vertical offset of the exciter coil
$n_{e}$	Number of excitation coil turns
$d_{e}$	Diameter of the excitation coil wire
$R_{e}$	Resistance of the excitation coil wire
$\sigma_{e}$	Electrical conductivity of the exciter coil
$\mu_{e}$	Magnetic permeability of the exciter coil
*d*	Plate thickness
σ	Electrical conductivity of pipe material
μ	Magnetic permeability of pipe material
$I_{e}$	Amplitude or the excitation current pulse
$Z_{dl}$	Load impedance connected to the detector coil

**Table 3:** Fixed parameters (constants) used for simulation.

Symbol	Value
$h_{e}=h_{d}$	10 mm
$lo_{e}=lo_{d}$	2 mm
$d_{e}=d_{d}$	0.315 mm
$\sigma_{e}=\sigma_{d}$	$5.998\times 10^{7}$ S/m for copper coils
$\mu_{e}=\mu_{d}$	$4\pi \times 10^{-7}$ H/m for copper coils
σ	$2.16\times 10^{6}$ S/m
μ	$\mu =\mu_{r}\mu_{0}$, $\mu_{r}=63$, $\mu_{0}=4\pi \times 10^{-7}$ H/m
$I_{e}$	200 mA
$R_{e}$	50 Ω
$Z_{dl}$	∞

**Table 4:** Estimated parameter values used to fabricate the PEC sensor.

Symbol	Value
$r_{di}$	25 mm
$r_{do}$	28 mm
$n_{d}$	300
$r_{ei}$	50 mm
$r_{eo}$	57 mm
$n_{e}$	600

**Table 5:** GP model accuracy on testing data, i.e., grey cast iron calibration blocks (with 2 mm lift-off).

Actual Thickness	Estimated Thickness	Absolute Error
7 mm	6.47 mm	0.53 mm
11 mm	10.50 mm	0.50 mm
15 mm	18.8 mm	3.8 mm
20 mm	25.2 mm	5.2 mm

**Table 6:** GP model accuracy on testing data, i.e., grey cast iron calibration blocks (with 14 mm lift-off).

Actual Thickness	Estimated Thickness	Absolute Error
7 mm	4.33 mm	2.67 mm
11 mm	9.99 mm	1.01 mm
15 mm	18.2 mm	3.2 mm
20 mm	25.1 mm	5.1 mm

## Supplementary Materials

The following are available online at http://www.mdpi.com/1424-8220/17/10/2208/s1.

## Abbreviations

The following abbreviations are used in this manuscript:

AWG	American Wire Gauge
EC	Eddy Current
EDM	Electric Discharge Machining
FEA	Finite Element Analysis
GP	Gaussian Process
NDE	Non-Destructive Evaluation
NDT	Non-Destructive Testing
PEC	Pulsed Eddy Current
PLA	Polylactide
PPMS	Physical Property Measurement System
